# Profiling of Both Dipeptidyl Peptidase III and Renin Reveals Higher Mortality in Septic Shock

**DOI:** 10.21203/rs.3.rs-9596404/v1

**Published:** 2026-05-21

**Authors:** Christopher L. Schaich, Mark C. Chappell, Shriram S. Mahabal, D Clark Files, Kendra N. Wright, Lane Smith, Ashish K. Khanna

**Affiliations:** Wake Forest University School of Medicine; Wake Forest University School of Medicine; Atrium Health Wake Forest Baptist Medical Center, Wake Forest University School of Medicine; Wake Forest University School of Medicine; Wake Forest University School of Medicine; Atrium Health Carolinas Medical Center, Wake Forest University School of Medicine; Wake Forest University School of Medicine

**Keywords:** Renin-angiotensin system, dipeptidyl peptidase III, renin, Angiotensin II, septic shock

## Abstract

Sepsis and septic shock are life-threatening conditions associated with high mortality and limited treatment options. Rapid identification of patients at risk for worse outcomes and/or targeted therapeutics are needed. Elevated levels of renin and dipeptidyl peptidase III (DPP3) individually have been associated with increased risk in septic shock, which may reflect an attenuated Angiotensin II response as DPP3 contributes to the metabolism of Angiotensin II. We applied an approach to distinguish both circulating renin and DPP3 concentrations at their clinically relevant threshold values for risk in septic shock patients of ≥ 180 pg/mL and ≥ 40 ng/mL, respectively. This analysis revealed four clinical phenotypes of patients with low risk (low renin/low DPP3), intermediate risk (high renin/low DPP3 or low renin/high DPP3) and those patients with the highest risk (high renin/high DPP3). We conclude that the subclassification of both circulating renin and DPP3 concentrations may better identify those patients at greater risk and inform more optimal treatment approaches.

## Introduction

Sepsis and septic shock mortality remains high (> 40%) among the millions of patients affected globally despite advances in therapy. Thus, rapid identification of patients at risk for worse outcomes and/or more specific treatment interventions are clearly needed. The most common circulating biomarker of sepsis severity is lactate, which is non-specific and has an inconsistent impact on prognosis and disease management [1]. The renin-angiotensin system (RAS) plays a critical role in the maintenance of blood pressure via generation of the vasoactive peptide Angiotensin II (Ang II) by the release of renin from the kidney to form Ang I followed by rapid cleavage by angiotensin converting enzyme (ACE) [2]. The activation of the RAS may occur early in sepsis in response to reduced blood pressure and evident by high levels of active renin although downstream components including Ang II, aldosterone and Angiotensin type 1 (AT_1_) receptor signaling may blunted [3–8]. In addition, emerging evidence suggests that dipeptidyl peptidase III (DPP3) is an important regulator of the RAS due to its ability to rapidly metabolize Ang II [8–10]. Dysfunction of the RAS, particularly alterations in Ang II generation and/or metabolism could contribute to worsening of blood pressure and inadequate tissue perfusion in critical illness including septic shock [4–8]. Recent studies find that elevated renin, particularly concentrations ≥ 180 pg/mL, associates with greater in-hospital mortality among patients with sepsis and may outperform lactate as a predictor of mortality in hypotensive critically ill patients [6, 8, 9–11]. In this case, high renin levels may reflect the loss of negative feedback on renin release by inappropriately low circulating Ang II and/or attenuated AT_1_R expression. Likewise, increased circulating levels of DPP3 in patients with severe sepsis or septic shock were associated with greater mortality, vasopressor requirement, and need for organ support [12–14]. We reported that septic patients with markedly elevated renin and DPP3 did not exhibit higher circulating Ang II or an elevated Ang II/Ang-(1–7) ratio further suggesting DPP3 involvement in Ang II metabolism [8]. Indeed, recent studies support a clinically relevant threshold for circulating DPP3 of 40 ng/mL in critical illness that correspond to the 97.5th percentile of the adult population (based on unpublished data from 5,021 participants in the Malmö Preventive Project) above which the risk of worse outcomes increases substantially [12–16]. Collectively, these reports suggest renin and DPP3 as easily obtainable biomarkers of septic shock severity. We hypothesized that profiling both renin and DPP3 may reveal a stronger association with mortality in septic shock and potentially inform optimal therapeutic interventions in these patients than either biomarker alone.

## Methods

The current study (Advocate Health Wake Forest University School of Medicine IRB 00111529, approved 05/07/2024) enrolled 50 adult patients, (convenience sample) after written informed consent and in accordance with the declaration of Helsinki. Patients were screened in the emergency rooms and intensive care units of Atrium Health Wake Forest Baptist Medical Center (Winston-Salem, NC, USA), Atrium Health High Point Medical Center (High Point, NC, USA) and Atrium Health Carolinas Medical Center (Charlotte, NC, USA). Inclusion criteria included adult patients with septic shock defined by the sepsis-3 criteria: evidence or suspicion of infection, serum lactate > 2 mmol/L and vasopressor use to maintain blood pressure. Blood samples were taken within the initial 12 hours of septic shock diagnosis with a median value of 5.8 [3.3–8.7] hours after initiation of vasopressor therapy. Blood was collected and allowed to clot for 90 mins at room temperature, centrifuged and the obtained serum stored at −80°C until assayed. Active renin protein was measured by a human renin ELISA (DRG International Fisher Scientific, Waltham, MA, USA) and DPP3 by a human DPP3 ELISA (LifeSpan Biosciences, Shirley, MA, USA) as previously described [8]. Patient characteristics were compared between patients stratified by DPP3 < 40 ng/mL and ≥ 40 ng/mL, and by renin < 180 pg/mL and ≥ 180 pg/mL based on cutpoints that appear to be clinically relevant in septic shock [6, 9, 12, 15, 16]. We used t-tests for normally distributed continuous variables, Kruskal-Wallis test for non-normally distributed continuous variables, and Fisher’s exact test for proportions. Associations of renin and DPP3 with 30-day mortality were assessed by Kaplan-Meier curves with log-rank test and hazard ratios (HR) from Cox proportional hazards models adjusted for age and sex. We separated patients into 4 groups corresponding to combinations of DPP3 and renin cutpoints and compared 30-day survival curves among the groups using log-rank test. Analyses were completed in R 4.2.3 (2023) and Prism 11.0 (GraphPad Software, San Diego, CA, USA) with a 2-sided α = 0.05.

## Results

**Figure 1:**
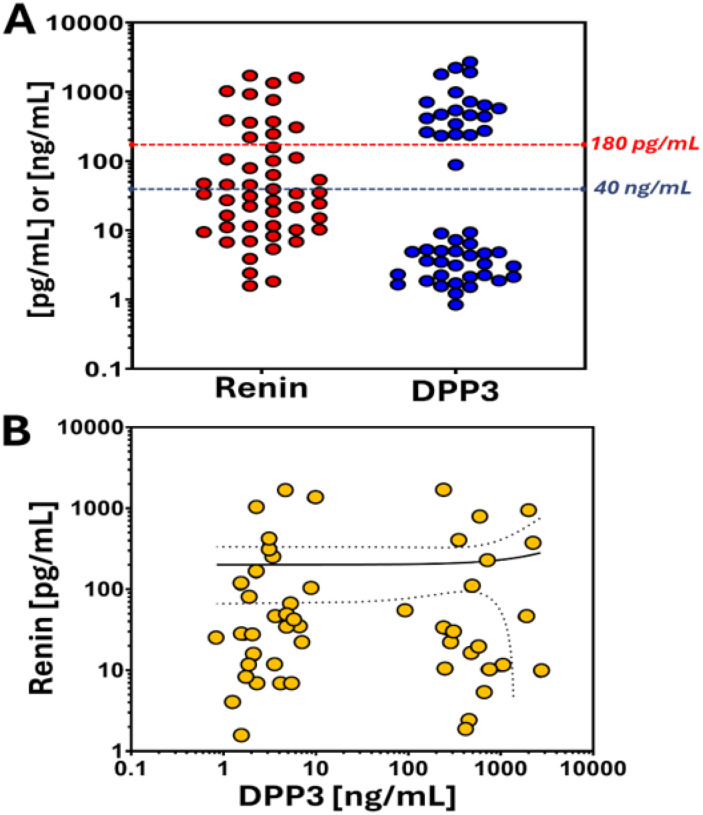
Distribution of Circulating Renin and DPP3 levels in septic shock. **A:** Distinct distribution of DPP3 among 2 populations [<40 ng/mL and ≥40 ng/mL] as compared to renin in septic shock patients. Dashed lines indicate proposed clinical cutoff values for renin (180 pg/mL) and DPP3 (40 ng/mL). **B:** Lack of association between circulating renin and DPP3 levels in septic shock patients (*R*^*2*^=*0.002; P*=*0.7592*).

**Figure 2:**
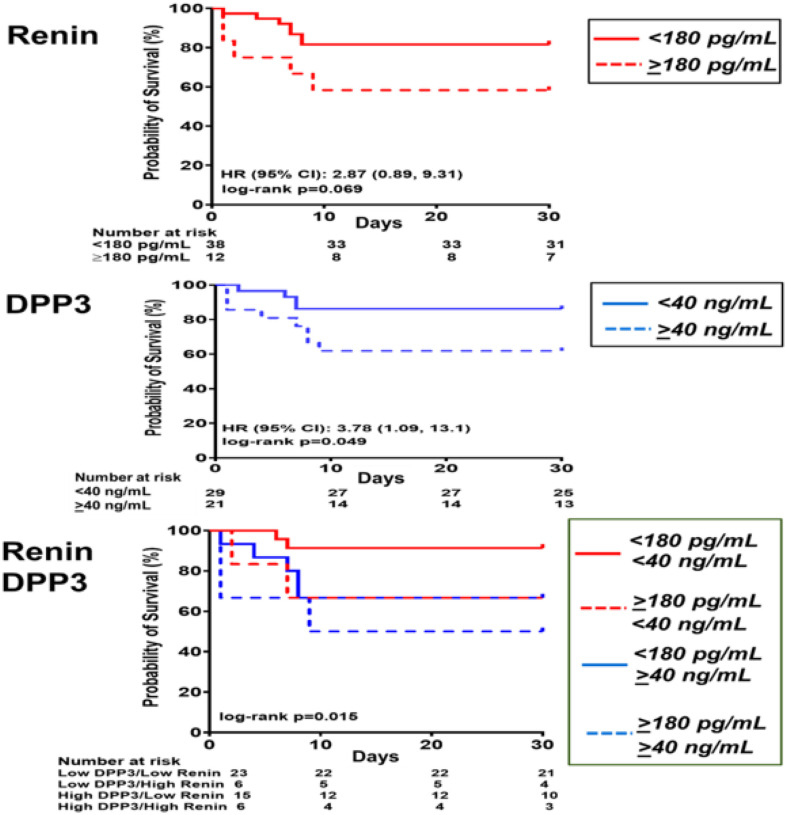
Comparison of Kaplan-Meier Survival Curves for Circulating Renin and DPP3. Estimates of the Kaplan-Meier survival curves reveal worse survival among patients with renin ≥180 pg/mL and DPP3 ≥40 ng/mL **(Renin/DPP3)**, and best survival among patients with lower levels of renin <180 pg/mL **(Renin)**, and DPP3 <40 pg/mL **(DPP3)**.

The overall distributions of renin and DPP3 in the serum of septic shock patients revealed a clear dichotomy for DPP3 but not renin (Fig. 1A). Moreover, there was no association between the circulating levels of renin and DPP3 (R^2^ = 0.002; P = 0.7592) in the total cohort of 50 patients (Fig. 1B). Patients with higher DPP3 (≥ 40 ng/mL; n = 21) or renin (≥ 180 pg/mL; n = 12) had significantly fewer vasopressor-, ventilator-, and dialysis-free days compared to those with lower DPP3 or renin, despite similar blood pressures and Sequential Organ Failure Assessment (SOFA) scores at time of blood draw **(Supplemental Table 1)**. Patients with renin ≥ 180 pg/mL tended to have worse 30-day mortality than participants with < 180 pg/mL (41.7% vs. 18.4%; log-rank p = 0.069; adjusted HR: 2.87, 95% CI: 0.89, 9.31; Fig. 2**-Renin)**. Similarly, those with DPP3 ≥ 40 ng/mL had worse 30-day mortality than participants with lower DPP3 (38.1% vs. 13.8%; log-rank p = 0.049; adjusted HR: 3.78, 95% CI: 1.09, 13.1; Fig. 2**-DPP3**). Finally, assigning participants to groups based on a combination of renin and DPP3 values revealed that the group with renin < 180 pg/mL and DPP3 < 40 ng/mL had the lowest 30-day mortality (8.7%) whereas patients with renin ≥ 180 pg/mL and DPP3 ≥ 40 ng/mL had the worst mortality (50%); groups with lower DPP3 but higher renin or higher DPP3 but lower renin had intermediate mortality (33% each; log-rank p = 0.015; Fig. 2**-Renin/DPP3**).

## Discussion

Adut patients with diagnosed septic shock in our prospective cohort demonstrated a strong association of increased DPP3 and renin with 30-day mortality with DPP3 having a higher hazards ratio for this outcome. Circulating DPP3 concentrations exhibited a unique bimodal distribution in this patient cohort while renin did not. When categorized into 4 plausible clinical phenotypes with high (> threshold cut points) and low/normal (< threshold cut points) combinations of each, a combination of low/normal DPP3 and renin had the best survival likelihood compared with high combinations of each, while a combination of high and low for either DPP3 or renin associated with intermediate risk. Indeed, our current results support previous studies that find either renin or DPP3 alone associates with poor outcomes in septic shock with each outperforming lactate [7–11, 12–16]. The analysis of both renin and DPP3, however, may provide a unique approach to more selectively identify patient groups that receive specific therapeutics at various time points in the course of the disease. Renin is formed and stored in secretory granules of the kidney and undergoes regulated release into the blood while DPP3 is a cytosolic peptidase expressed in many tissues and release appears to occur following cellular injury [9, 16]. DPP3 sequentially cleaves the Aspartate^1^-Arginine^2^ dipeptide from the N-terminus of Ang II to form Ang-(3–8) (Ang IV), and more rapidly splits off Valine^3^-Tyrosine^4^ to generate the biologically inactive peptide Ang-(5–8). DPP3 also hydrolyzes the same dipeptides from Ang-(1–7) to form Ang-(3–7) and further to Ang-(5–7), although the role of DPP3-dependent metabolism of Ang-(1–7) in septic shock is presently unclear [17]. Our results are comparable to other reports that have individually shown both DPP3 and renin associated with poor outcomes in septic shock with each outperforming lactate; however, little is known about the interplay of elevated DPP3 with the classic and alternate RAS in septic shock. Procizumab, a monoclonal antibody to DPP3 that inhibits enzyme activity, improved outcomes in preclinical models of septic shock and in a preliminary study of septic shock patients with high DPP3 enzymatic activities [18–20]. The clinical use of the DPP3 antibody is currently in early phase human trials. Exogenous treatment with Ang II decreases the elevated renin levels in patients with septic shock and in a post hoc analysis of the ATHOS-3 trial, Ang II treatment associated with a survival benefit in those with renin concentrations higher than the population median (173 pg/ml) [9]. The grouping of septic shock patients into different categories of DPP3 and renin concentrations may provide an opportunity to tailor these specific therapeutics to each of these patient types at various time points in the disease course. We acknowledge our study is limited by sample size and a disproportionate patient distribution in the different concentrations of renin and DPP3 subclasses, as well as a one-time sampling of each patient. As septic shock is a dynamic process with rapid changes over time, repeated measures over extended time periods in a larger cohort are required to clearly establish meaningful patterns. Nonetheless, subclassification of both circulating levels of renin and DPP3 may begin to clarify the complex role of DPP3 and the RAS response, provide a more rapid indication of risk and better inform therapies that target these systems in critically ill patients.

## Supplementary Files

This is a list of supplementary files associated with this preprint. Click to download.

- 05012026DPP3Supplement.docx

## Abbreviations

ACE: angiotensin converting enzyme
Ang II: angiotensin II
ELISA: enzyme-linked immunosorbent assay
DPP3: dipeptidyl peptidase III
RAS: renin angiotensin system
ATHOS3: Angiotensin II in High Output Shock
SOFA: sequential organ failure assessment
